# Nanofused Hierarchically Porous MIL-101(Cr) for Enhanced Methyl Orange Removal and Improved Catalytic Activity

**DOI:** 10.3390/ma15103645

**Published:** 2022-05-19

**Authors:** Minmin Zou, Ming Dong, Mingliang Luo, Hexin Zhu, Tian Zhao

**Affiliations:** School of Packaging and Materials Engineering, Hunan University of Technology, Zhuzhou 412007, China; zz09181002@163.com (M.Z.); dongming666@126.com (M.D.); westbrook123666@163.com (M.L.); zhx19a@126.com (H.Z.)

**Keywords:** hierarchically porous, MIL-101(Cr), butyric acid, MO adsorption, catalysis

## Abstract

Hierarchically porous MIL-101(Cr) (H-MIL-101(Cr)) with meso/macro-pores was directly prepared via nanofusion progress by using butyric acid as a modulating agent. In the methyl orange (MO) adsorption experiments, H-MIL-101(Cr) showed a high adsorption capability of 369.8 mg g^−1^, which was 1.52-fold greater than that of pristine MIL-101(Cr) (P-MIL-101(Cr)). While in the oxidation reaction of indene and 1-dodecene tests, H-MIL-101(Cr) presented much higher catalytic efficiency, with turnover frequency (TOF) values of 0.7242 mmol g^−1^ min^−1^ and 0.1492 mmol g^−1^ min^−1^, respectively, which were 28% and 34% greater than that in the case of P-MIL-101(Cr). Thus, compared with P-MIL-101(Cr), H-MIL-101(Cr) exhibited better removal efficiency and higher levels of activity in the oxidation reactions of indene and 1-dodecene. The unique structure of H-MIL-101(Cr) also contributed to its superior performance in these processes.

## 1. Introduction

Metal-organic frameworks (MOFs) are a class of crystalline porous materials composed of metal ions or metal clusters and organic ligands [1,2]. MOFs have a unique advantage over conventional porous materials in that they have a wide selection of metal centers and a wide variety of organic ligands [3,4]. MOFs have high specific surface areas, controllable pore sizes and special active sites [5,6,7]. Hence, they are used in a great variety of applications in drug delivery, adsorption, catalysis, separation, sensors, etc. [8,9,10,11,12].

MIL-101(Cr) is one of the widely studied classical Cr-based MOFs. It is a three-dimensional porous material with chromium ion as the metal center and terephthalic acid as the organic ligand, with the chemical formula [Cr_3_(O)X(bdc)_3_(H_2_O)_2_] (bdc = terephthalate, X = OH or F) [13]. MIL-101(Cr) has better physicochemical properties and chemical stability than other MOFs (e.g., MOF-5 and HKUST-1), making it an ideal adsorbent with good environmental responsiveness [14,15,16]. Furthermore, MIL-101(Cr) has crystalline water molecules at the end of its molecular structure, which can be removed under heating conditions to produce Lewis acid sites. Therefore, MIL-101 has remarkable stability to water. MIL-101 has good applications in the adsorption and removal of antibiotic drugs, organic dyes and heavy metal ions such as lead and mercury from water bodies [17,18,19,20].

In recent years, with rapid industrial development, a large number of organic dyeing solutions are used in the textile, tannery and paper industries, and some organic dyes are discharged into the water environment without thorough treatment, which can cause great pollution to the environment [21,22,23]. Meanwhile, the extensive use of hair dyes and luminescent solar concentrator (LSC) technology also contributes to the discharge of residual hair dyes and fluorescent dyes, which aggravate the pollution of the water environment [24,25]. Methyl Orange (MO) is one of the widely used azo dyestuffs, especially in printing and dyeing textiles, which will cause a lot of pollution to the environment if discharged into the water environment under incomplete treatment. MO molecules could penetrate biological cells, destroy and change the genetic material of organisms and also enter the human body through other seafood such as fish and shrimp, endangering human health [26]. Thus, it is necessary to remove MO from wastewater. MIL-101(Cr) has good adsorption performance, so it will have a good potential application in the adsorption of organic dyes in wastewater.

Pristine MIL-101(Cr) (P-MIL-101(Cr)) has two mesopores with diameters of 2.9 nm and 3.4 nm, respectively, and a maximum pore window of approximately 1.6 nm [27,28]. However, the small pores are not conducive to mass transfer, which seriously limits the application of MIL-101(Cr), especially in the case of large molecules [29,30]. Due to the high thermal/chemical stability of MIL-101(Cr), it is not easy to prepare hierarchically porous MIL-101(Cr) (H-MIL-101(Cr)). In the previous report, MIL-101(Cr) is often combined with other materials to fabricate hierarchically porous structure as composites [31,32,33,34]. On the contrary, pure H-MIL-101(Cr) is still rarely reported.

Here, we presented a nanofusion method using butyric acid as a modulator, by which meso/macroporous MIL-101(Cr) was synthesized. Compared with the conventional method to synthesize MIL-101(Cr), the H-MIL-101(Cr) synthesized by this methodology possessed additional meso/macropores with diameters around 30~100 nm, which are formed by nanofused MIL-101(Cr) crystals, in addition to the natural pores of MIL-101(Cr). For the adsorption of MO in an aqueous solution, the adsorption capacity of H-MIL-101(Cr) was 1.52 times higher than that of P-MIL-101(Cr). MIL-101(Cr) can be used as a catalyst in the oxidation reaction of indene and 1-dodecene. The catalytic efficiency was best at a MIL-101(Cr) addition of 10 mg, while H-MIL-101(Cr) exhibited higher catalytic activity—more than 28% and 32% higher than P-MIL-101(Cr), respectively.

## 2. Experimental

### 2.1. Raw Materials and Reagents

The raw materials and reagents involved in this paper are listed below: chromium (III) nitrate nonahydrate (Cr(NO_3_)_3_·9H_2_O, 99%, Aladdin, Shanghai, China), benzene-1,4-dicarboxylic acid (H_2_BDC, 99%, Aladdin, Shanghai, China), butyric acid (99%, Aladdin, Shanghai, China), N, N-Dimethylformamide (DMF, 99.5%, AR, Aladdin, Shanghai, China) and ethanol (99.7%, AR, Aladdin, Shanghai, China). The above materials and reagents were used directly without further processing.

### 2.2. Synthesis and Purification of H-MIL-101(Cr) and P-MIL-101(Cr)

H-MIL-101(Cr): 0.4 g Cr(NO_3_)_3_·9H_2_O (1 mmol) and 0.166 g H_2_BDC (1 mmol) were mixed well with 3 mmol butyric acid in 5 mL deionized water and stirred magnetically for 30 min. After stirring, the mixture was quickly transferred to a 25 mL polytetrafluoroethylene (PTFE, Youmai, Shanghai, China) autoclave and sealed. The autoclave was placed in the reactor, and the temperature was set at 220 °C for 8 h. After the reaction, the autoclave was allowed to cool naturally.

The green product was collected by centrifugation and then dispersed in 5 mL DMF and sonicated at 80 °C for 1 h. The product was again separated by centrifugation from DMF, uniformly dispersed in 5 mL ethanol and sonicated at 80 °C for 1 h. Separate by centrifugation in a centrifuge and collect the final green product. The purified green crystals were dried in a vacuum oven at 120 °C for 2 h.

P-MIL-101(Cr): 0.4 g of Cr(NO_3_)_3_·9H_2_O (1 mmol) and 0.166 g of H_2_BDC (1 mmol) were mixed in 5 mL of deionized water and stirred for 30 min. The mixture was then transferred to a 25 mL PTFE autoclave. The reaction condition and treatment procedure that were followed were the same as those described above.

### 2.3. Characterization

(1) The samples were tested with a powder X-ray diffractometer (D8 Advance, Bruker, Karlsruhe, Germany) using a Cu target, a Kα radiation (λ = 1.54182 nm) light source, a set operating voltage of 30 kV, a test angle range of 5° to 80° and a test time of 30 min.
(2) The specific surface area and pore volume of the samples were determined by physical absorptiometry (NOVA-4200e, Quantachrome, Boynton Beach, FL, USA), and all samples were pretreated in the same way before testing. The pressure range of the test was 10.1325~101.325 kPa, high purity N_2_ was used as the adsorbent and the test temperature was −196 °C.
(3) The microscopic morphology and particle size of the samples were characterized by transmission electron microscopy (Talos F200X, FEI, Hillsboro, OR, USA), and the samples should be pretreated before conducting scanning. The samples were dispersed in ethanol and placed on a common copper grid.
(4) Scanning electron microscopy (Nova NanoSEM230, FEI, Hillsboro, OR, USA) was used for characterizing the crystal morphology structure of the samples. The sample is placed on conductive adhesive and sprayed with gold.
(5) A UV-Vis spectrophotometer (UV-2600, Shimadzu, Suzhou, China) was used to test the concentration of dyes before and after the adsorption of the adsorbent.
(6) The oxidation reaction of indene and 1-dodecene tests was monitored in real time using gas chromatography (7890A, Agilent, Beijing, China) at room temperature. The concentration of the oxidation reaction products was determined by using dodecane as a reference.

### 2.4. Methyl Orange Adsorption Experiments

A total of 5 mg of adsorbent (H-MIL-101(Cr) or P-MIL-101(Cr)) was added to 50 mL of MO solution (50 mg L^−1^~500 mg L^−1^) until the adsorption equilibrium was reached (the adsorption time was over 24 h). Take an appropriate amount of supernatant before and after adsorption and measure the absorbance of the solution by using a UV spectrophotometer. The adsorption capacity *q_e_* (mg g^−1^) was calculated according to Equation (1) [35].

(1) $$q_{e}=\frac{V\left(C_{0}-C_{e}\right)}{m}$$

where *C*_0_ (mg L^−1^) is the concentration of the initial MO solution, *C_e_* (mg L^−1^) is the concentration of MO in the solution at the adsorption equilibrium, *V* (L) is the volume of the MO solution used in the adsorption experiment and *m* (g) is the weight of the adsorbent used in the experiment.

### 2.5. Adsorption Kinetics and Isothermal Adsorption Model

1. Adsorption kinetic model

In order to study the adsorption process of MO by adsorbent, the adsorption data were fitted to quasi-primary and quasi-secondary kinetics, respectively. The equations for the pseudo-first-order kinetic and the pseudo-second-order kinetic and the expressions for the intra-particle diffusion (Weber–Morris) model are shown in Equations (2)–(4), respectively [36,37,38].

(2) $$ln(q_{e}-q_{t})=lnq_{e}-k_{1}t$$

(3) $$\frac{t}{q_{t}}=\frac{1}{k_{2}q_{e}^{2}}+\frac{t}{q_{e}}$$

(4) $$q_{t}=k_{p}t^{0.5}+I$$

where *q_e_* and *q_t_* are the adsorption amounts (mg g^−1^) at the adsorption equilibrium and at time *t*, *t* is the adsorption time (min), *I* is the parameter and *k*_1_ (min^−1^), *k*_2_ (g mg^−1^ min^−1^) and *k_p_* (mg g^−1^ min^−0.5^) are the rate constants for the above model, respectively.

2. Isothermal adsorption model
(1) Langmuir model

The Langmuir model assumes that the adsorption process of the adsorbent on the target adsorbate is a single molecular layer surface adsorption and that all adsorption sites are identical [39]. The expression of the Langmuir isothermal adsorption model is shown in Equation (5) [40].

(5) $$\frac{C_{e}}{q_{e}}=\frac{C_{e}}{q_{m}}+\frac{1}{k_{L}q_{m}}$$

where *C_e_* is the concentration of the solution at the adsorption equilibrium (mg L^−1^), *q_m_* is the saturation adsorption amount (mg g^−1^), *q_e_* is the amount of adsorbent adsorbed at the adsorption equilibrium (mg g^−1^) and *K_L_* is the adsorption equilibrium constant (L mg^−1^). A larger *K_L_* value indicates a stronger adsorption capacity.

(2) Freundlich model

The Freundlich model assumes that the adsorbent surface energy is not uniform and that there is multilayer adsorption of target ions on the adsorbent surface instead of simple single molecular layer adsorption [41]. The expression of the Freundlich isothermal adsorption model is shown in Equation (6) [42].

(6) $$lnq_{e}=lnK_{F}+\frac{1}{n}lnC_{e}$$

where *C_e_* is the concentration of adsorbate in the solution at the adsorption equilibrium (mg L^−1^), *q_e_* is the amount of adsorbent adsorbed at the adsorption equilibrium (mg g^−1^), *K_F_* is the Freundlich equilibrium constant (mg g^−1^) and 1/*n* is the parameter.

(3) Temkin model

The Temkin model hypothesizes that the adsorbent surface heat of adsorption shows a linear decreasing trend with increasing coverage [43].

(7) $$q_{e}=Bln\left(A\right)+BlnC_{e}$$

where *q_e_* is the amount of adsorbent adsorbed at the adsorption equilibrium (mg g^−1^), *C_e_* is the concentration of adsorbate in the solution at the adsorption equilibrium (mg L^−1^), *B* is the physical quantity related to the heat of adsorption and *A* is the equilibrium constant (L g^−1^) of the Temkin isothermal adsorption model.

### 2.6. Analysis of Catalytic Activity

Indene oxidation reaction: 10 mg of catalyst (P-MIL-101(Cr) or H-MIL-101(Cr)), 0.5 mmol of indene and 2 mL of CH_3_CN were homogeneously mixed in the apparatus. The mixture was then spiked at 70 °C for 10 min. Add 500 uL of H_2_O_2_ (30 wt%, 5 mmol) to the mixture quickly, at which point the reaction begins. The reaction was detected in real time though gas chromatography (GC), and the catalyst intermediates were collected at different reaction times.

1-dodecene oxidation reaction: 10 mg of catalyst (P-MIL-101(Cr) or H-MIL-101(Cr)) and 0.5 mmol of 1-dodecene were dissolved in 2 mL of CH_3_CN solution and spiked at 70 °C for 10 min in a closed glass vessel. Then, 500 uL of H_2_O_2_ (30 wt%, 5 mmol) was added rapidly, at which point the reaction started. The reaction was detected in real time via GC.

## 3. Results and Discussion

**Figure 1:**
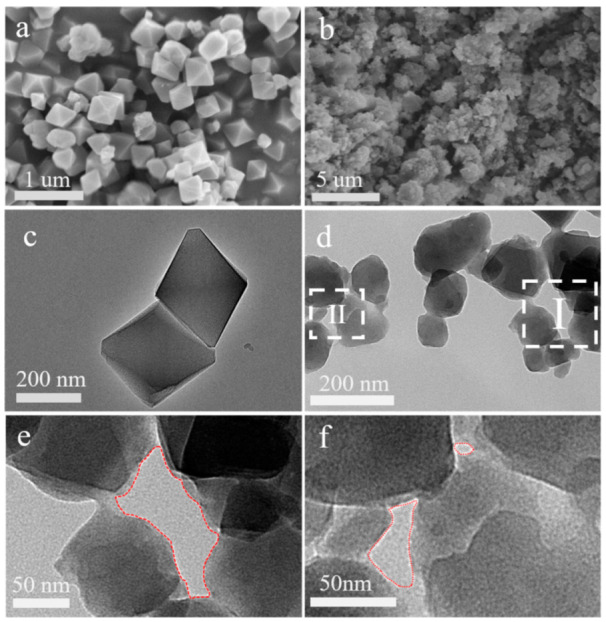
SEM images of (**a**) P-MIL-101(Cr) and (**b**) H-MIL-101(Cr), TEM images of (**c**) P-MIL-101(Cr) and (**d**) H-MIL-101(Cr), (**e**,**f**) amplification regions of I and II in (**d**), respectively.

The crystal morphologies of H-MIL-101(Cr) and P-MIL-101(Cr) are shown in Figure 1. Unlike the pristine reported MIL-101(Cr) morphology with well dispersed crystals [44,45,46], H-MIL-101(Cr) showed aggregates of nanocrystals with irregular shapes, and the edges of nanocrystals were fused together to form extra larger meso/macropores (Figure 1b). Transmission electron microscopy (TEM) images clearly show the presence of abundant nanofusion between nanoscale H-MIL-101(Cr) crystals and crystals (Figure 1d). Due to the nanofusion of adjacent nanoparticles, extra meso/macropores with pore sizes around 30~100 nm were formed (Figure 1e,f). In contrast, the P-MIL-101(Cr) crystals synthesized by the conventional method showed an octahedral structure with a dispersed distribution of crystal particles and no crystal nanofusion phenomenon, which is consistent with the results of the SEM and TEM images (Figure 1a,c). According to the particle size statistics, P-MIL-101(Cr) has a greater particle size of ~208 nm compared to H-MIL-101(Cr) (Figure S1).

**Figure 2:**
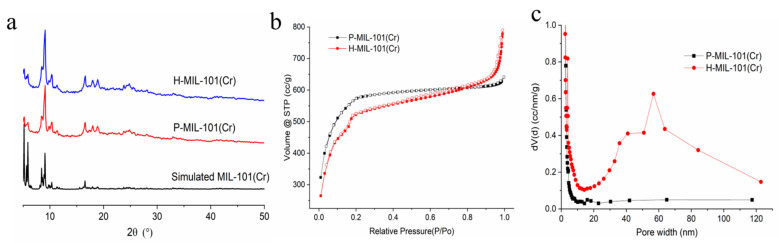
(**a**) Small-angle PXRD patterns of H-MIL101(Cr) and P-MIL-101(Cr), (**b**) nitrogen sorption isotherms of H-MIL-101(Cr) and P-MIL-101(Cr), (**c**) BJH pore size map of H-MIL101(Cr) and P-MIL-101(Cr).

Powder X-ray diffraction (PXRD) tests were performed on samples H-MIL-101(Cr) and P-MIL-101(Cr), and the results are shown in Figure 2a. The obtained PXRD patterns of H-MIL-101(Cr) and P-MIL-101(Cr) corresponded to the simulated characteristic MIL-101(Cr) pattern, thus indicating that both P-MIL-101(Cr) and H-MIL-101(Cr) were pure and well-crystallized target products (Figure 2a).

Figure 2b showed the N_2_ adsorption–desorption curves of P-MIL-101(Cr) and H-MIL-101(Cr). It can be demonstrated that there is no hysteresis return line between the adsorption and desorption curves of P-MIL-101(Cr), which belongs to the type I(b) adsorption isotherm and is a typical adsorption curve of MIL-101(Cr) [47,48,49]. In contrast, for H-MIL-101(Cr), there was a significant hysteresis curve back to the N_2_ adsorption detoxification curve in the range of P/P_0_ = 0.45~0.99. This suggested the presence of meso/macropores in H-MIL-101(Cr) [50]. The pore size distribution (PSD) curves of P-MIL-101(Cr) and H-MIL-101(Cr) using the Barrett–Joyner–Halenda (BJH) model were also displayed in Figure 2c. The results indicated that P-MIL-101(Cr) only had the small intrinsic mesopores, while H-MIL-101(Cr) not only possessed intrinsic mesopores but also possessed much larger meso/macropores with pore sizes ranging from 30 to 100 nm, which was perfectly in line with the electron microscopic results. The other porous information can be found in Table 1.

**Figure 3:**
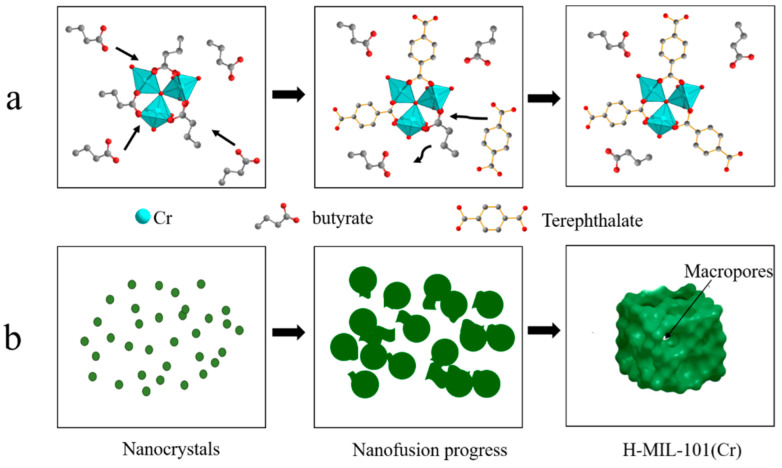
Diagram of the mechanism of H-MIL-101(Cr) synthesis using butyric acid. (**a**) Substitution of terephthalate ligands for butyrate. (**b**) Nanofusion process of the crystal nucleus.

A plausible mechanism of the formation of H-MIL-101(Cr) is illustrated below (Figure 3). The experiment started with the first coordination of excess butyric acid with Cr^3+^ to produce the intermediate product (butyric acid-chromium complex) (Figure 3a). As the reaction proceeded and the reaction temperature increased, H_2_bdc started to dissolve, and bdc^2−^ slowly replaced the butyrate of the intermediate product, thus forming the MIL framework structure (Figure 3a). The fast rate of nucleation during this process and the limited number of Cr^3+^ and bdc^2−^ led to a restricted skeleton growth per core, resulting in a very small crystal size of MIL-101(Cr). As the reaction continues, the nanoparticles agglomerate to form mesopores or even macropores, at which time the interfacial fusion of nanoparticles occurs (Figure 1d–f and Figure 3b).

**Figure 4:**
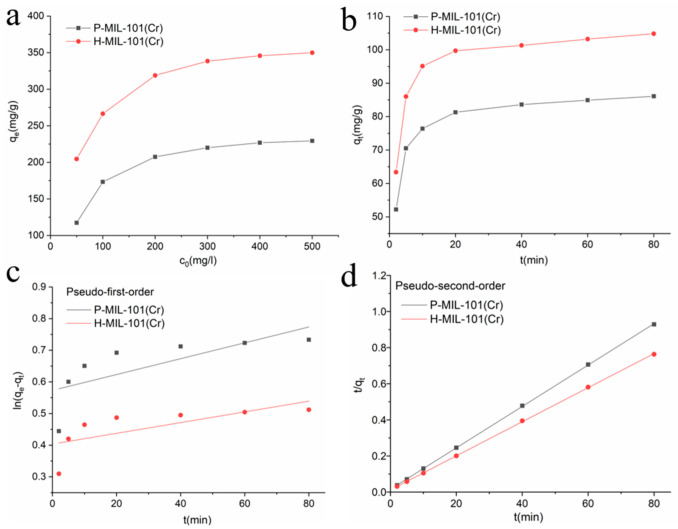
(**a**) The adsorption capacities of MILs with variable concentrations of MO. The uptake of MO was assessed by dispersing 5 mg MILs in aqueous MO liquids (50 mg L^−1^ to 500 mg L^−1^) for 24 h at room temperature (25 °C). (**b**) Effect of adsorption time on the adsorption of MO by MILs. (**c**) Pseudo-first-order and (**d**) pseudo-second-order of the kinetics data.

Here, MO was chosen as the model dye for the adsorption experiments (see Figure S2 for the molecular structure of MO). The effect of the initial solution pH on the adsorption of MO by P-MIL-101 was examined (Figure S3). At a pH of 7, P-MIL-101 had the most optimal adsorption capacity. Therefore, adsorption kinetics and isotherm measurement experiments were performed at pH = 7. According to Table 1, the BET surface areas of P-MIL-101(Cr) and H-MIL-101(Cr) were 1650 m^2^ g^−1^ and 1742 m^2^ g^−1^, respectively. Nevertheless, for MO adsorption, H-MIL-101(Cr) exhibited a high adsorption capacity of 369.8 mg g^−1^, which was 1.52-fold higher than that of P-MIL-101(Cr) (229.4 mg g^−1^) (Figure 4a). Compared with the other adsorbents, the adsorption value of H-MIL-101(Cr) toward MO was significantly high (Table S1) [51,52,53,54,55,56,57,58,59,60,61,62]. This may be attributed to its unique meso/macroporous structure, which allowed more big dye molecules, such as MO, into the larger meso/macropores and hence improved its dye adsorption capability.

The adsorption capacities of P-MIL-101(Cr) and H-MIL-101(Cr), with various initial concentrations of MO (50 mg L^−1^~500 mg L^−1^), are displayed in Figure 4a. The adsorption curves of P-MIL-101(Cr) and H-MIL-101(Cr) became flatter and flatter as the initial concentration of MO increased. The time-variable adsorption capacities of P-MIL-101(Cr) and H-MIL-101(Cr) are presented in Figure 4b. The adsorption rate of MILs for MO was quite high within the first 20 min, and then the increase in the adsorption amount became gradually smaller with increasing time. The results of the adsorption kinetic analysis of the adsorption behavior of the samples are shown in Figure 4c,d and Table S2. The correlation coefficients (R^2^) of the pseudo-second-order kinetic models for both P-MIL-101(Cr) and H-MIL-101(Cr) were 0.999, far greater than that of the pseudo-first-order kinetic model. The theoretical adsorption amount was very close to the experimental results, indicating that the adsorption processes of P-MIL-101(Cr) and H-MIL-101(Cr) on MO were consistent with pseudo-second-order kinetics, and the interaction between the adsorbent and the ion was mainly chemisorption.

**Figure 5:**
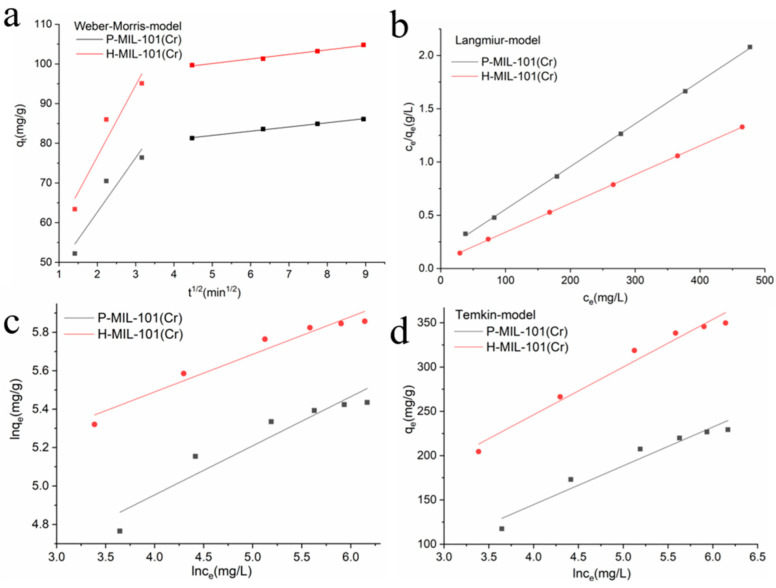
Fitting results of MO on MILs by using the (**a**) Weber-Morris, (**b**) Langmuir, (**c**) Freundlich and (**d**) Temkin models.

The Weber–Morris model was also employed to fit the adsorption process of the sample on MO (Figure 5a). The linear fitting equations for P-MIL-101(Cr) and H-MIL-101(Cr) consisted of two line segments with different slopes. It indicated that the adsorption rates of P-MIL-101(Cr) and H-MIL-101(Cr) toward MO were controlled by both liquid film diffusion and internal diffusion, and the adsorption process was not only controlled by a single process. The adsorption data of P-MIL-101(Cr) and H-MIL-101(Cr) were fitted to the adsorption isotherm models Langmuir, Freundlich and Temkin, and the results are shown in Figure 5b–d and Table S2. The Langmuir model presented an excellent correlation, with a correlation coefficient R^2^ > 0.999 for both samples, and the maximum adsorption amount of MO was very close to the calculated theoretical adsorption value (Table S2). For the Freundlich and Temkin models, the correlations were much lower, with correlation coefficients R^2^ of 0.885 and 0.935, respectively (Table S2). Thus, the Langmuir model could more accurately describe the adsorption process of P-MIL-101(Cr) and H-MIL-101(Cr) on MO, and the adsorption was mainly monolayer adsorption.

In order to further illustrate the advantages of the hierarchically porous structure of MIL-101(Cr), two oxidation reactions were employed to test the catalytic activity of the samples. The indene (Runs 1, 2) and 1-dodecene (Runs 3, 4) can be oxidized to their respective carboxylic acids by H_2_O_2_ in CH_3_CN with the presence of MIL-101(Cr) (Figure S4) [63]. The experimental results of the catalytic oxidation reactions of P-MIL-101(Cr) and H-MIL-101(Cr) are shown in Table 2. The conversion of indene and 1-dodecene was increasing with the increase of P-MIL-101(Cr) and H-MIL-101(Cr) additions. At the addition of P-MIL-101(Cr) and H-MIL-101(Cr) of 10 mg, the conversion of indene and 1-dodecene had basically reached the optimal state. A subsequent increase in the mass of P-MIL-101(Cr) and H-MIL-101(Cr) resulted in very little change in the conversion rate, so the catalytic effect was optimal at the addition of P-MIL-101(Cr) and H-MIL-101(Cr) at 10 mg. The catalytic activities of P-MIL-101(Cr) and H-MIL-101(Cr) were investigated at a catalyst content of 10 mg, and the results are shown in Figures S5 and S6. Obviously, H-MIL-101(Cr) demonstrated higher catalytic activity than that of P-MIL-101(Cr) (Table 2, Figures S5 and S6). For instance, in the 1-dodecene oxidation, the conversion rate of 1-dodecene was nearly 90% for H-MIL-101(Cr) at 60 min, while in the case of P-MIL-101(Cr), the conversion rate was only 64% (Figure S5). Regarding the oxidation of indene, although the conversion of P-MIL-101(Cr) and H-MIL-101(Cr) catalyzed indene oxidation ended up being 100%, it can be demonstrated that H-MIL-101(Cr) catalyzed a higher conversion than P-MIL-101(Cr), especially at 60 min, where the difference in conversion was close to 20% (Figure S6). Therefore, the conversion frequency (TOF) values of H-MIL-101(Cr) were significantly better than those of P-MIL-101(Cr) (Table 2). In 1-dodecene oxidation, the TOF value of H-MIL-101(Cr) was 1.34 times (0.1492 vs. 0.1108 mmol g^−1^ min^−1^) higher than that of P-MIL-101(Cr). Similarly, in ninhydrin oxidation, the TOF value of H-MIL-101(Cr) was 1.28 times (0.7242 vs. 0.5641 mmol g^−1^ min^−1^ at 60 min) larger. Absolutely, the hierarchical porosity of H-MIL-101(Cr) promoted the transport of both reactants and products in the above reactions. Thus, under the same conditions, H-MIL-101(Cr) possessed higher catalytic activity than P-MIL-101(Cr). Furthermore, after three cycles, H-MIL-101(Cr) still had relatively good catalytic activity, while for P-MIL-101(Cr), the conversion of the reactants of both oxidations was below 40%, indicating that the cyclability of P-MIL-101(Cr) was much lower than that of H-MIL-101(Cr) (Figures S5 and S6).

## 4. Conclusions

In conclusion, H-MIL-101(Cr) can be facially synthesized via the nanofusion method with the presence of butyric acid. The prepared H-MIL-101(Cr) possessed a meso/macroporous structure, exhibiting much better adsorption capability toward harmful dye MO and exhibiting excellent catalytic performance in catalytic oxidation reactions compared with the control P-MIL-101(Cr).

## Figures and Tables

**Table 1:** Particle size, surface area and pore volume of samples.

Sample	Particle Size /(nm) ^a^	*S*_BET_ /(m^2^ g^−1^) ^b^	*S*_Langmuir_ /(m^2^ g^−1^)	*V*_pore_ /(cm^3^ g^−1^) ^c^
P-MIL-101(Cr)	208 (1.77)	1742	2762	1.40
H-MIL-101(Cr)	97 (4.39)	1650	2698	1.02

^a^ The mean particle size acquired from Gaussian model statistics, with values in parentheses as standard deviations. ^b^ Calculated specific surface area in the pressure range 0.05 < P/P_0_ < 0.2 from the N_2_ adsorption isotherm, with a calculated standard deviation of ±50 m^2^ g^−1^, approximately. ^c^ Calculated from N_2_ sorption isotherms at 77 K (P/P_0_ = 0.95) for pores ≤ 20 nm.

**Table 2:** Summary on catalytic reactions.

Run	Sample ^a^	Addition (mg)	Reaction	TOF (mmol g^−1^ min^−1^) ^c^	Conversion (%)
1	P-MIL-101(Cr)	0	Indene oxidation reaction ^b^	0	0
		5		0.8967	53.8
		10		0.5641	67.7
		15		0.3833	69.0
		20		0.2892	69.4
2	H-MIL-101(Cr)	0		0	0
		5		1.185	71.1
		10		0.7242	86.9
		15		0.4905	88.3
		20		0.3704	88.9
3	P-MIL-101(Cr)	0	1-Dodecene oxidation reaction ^b^	0	0
		5		0.1607	48.2
		10		0.1108	64.5
		15		0.0722	65.2
		20		0.05475	65.7
4	H-MIL-101(Cr)	0		0	0
		5		0.2453	73.6
		10		0.1492	89.5
		15		0.9978	89.8
		20		0.075	90.1

^a^ Samples were dried overnight prior to the experiment. ^b^ Molar proportion of materials: (indene or 1-dodecatriene)/H_2_O_2_ = 4:1, and the reaction was carried out at 70 °C. ^c^ The TOF value is equal to the ratio between the molar conversion of the raw material to the product of the mass of MILs and the reaction time.

## Supplementary Materials

The following supporting information can be downloaded at: https://www.mdpi.com/article/10.3390/ma15103645/s1, Figure S1: Particle size distribution of P-MIL-101(Cr) (a) and H-MIL-101(Cr) (b); Figure S2: Dye molecule diagram of MO; Figure S3: Effect of pH on the adsorption of MO by P-MIL-101; Figure S4: Reaction on scheme for (a) indene oxidation reaction, (b) 1-dodecene oxidation; Figure S5: (a) Time-dependent conversion of 1-dodecene by P-MIL-101(Cr) and H-MIL-101(Cr). (b) Comparison of the conversion for P-MIL-101(Cr) and H-MIL-101(Cr) over three reaction runs; the reaction time is 20 min; Figure S6: (a) Time-dependent conversion of indene by P-MIL-101(Cr) and H-MIL-101(Cr). (b) Comparison of the conversion for P-MIL-101(Cr) and H-MIL-101(Cr) over three reaction runs; the reaction time is 20 min; Table S1: Adsorption capacities (*q_max_*) of MO on several adsorbents; Table S2: Characteristic parameters of the adsorption of dyes on the samples.
