# Supplementary material for: Nanofused Hierarchically Porous MIL-101(Cr) for Enhanced Methyl Orange Removal and Improved Catalytic Activity
Source: Materials (Basel). 2022 May 19;15(10):3645. doi: 10.3390/ma15103645 (PMC9146841; doi:10.3390/ma15103645)
Supplement: Supplementary file 1 [file materials-15-03645-s001.zip › materials-1715352-supplementary.pdf]

Nanofused hierarchically porous MIL-101(Cr) for enhanced methyl orange removal and improved catalytic activity

Minmin Zou, Ming Dong, Mingliang Luo, Hexin Zhu, Tian Zhao*

School of Packaging and Materials Engineering, Hunan University of Technology, Zhuzhou 412007, China

Abstract: Hierarchically porous MIL-101(Cr) (H-MIL-101(Cr)) with meso/macro-pores was directly prepared via nanofusion progress by using butyric acid as a modulating agent. In the methyl orange (MO) adsorption experiments, H-MIL-101(Cr) showed a high adsorption capability of 369.8 mg g^{-1}, which was 1.52 folds than that of pristine MIL-101(Cr) (P-MIL-101(Cr)). While in the oxidation reaction of indene and 1-dodecene tests, H-MIL-101(Cr) presented much higher catalytic activity with the turnover frequency (TOF) value of 0.7242 mmol g^{-1} min^{-1} and 0.1492 mmol g^{-1} min^{-1}, respectively, which was over 28 % and 34 % than that of in the case of P-MIL-101(Cr). Thus, compared with the P-MIL-101(Cr), H-MIL-101(Cr) demonstrated quite good removal efficiency toward MO and exhibited much better catalytic activity in the oxidation reaction of indene and 1-dodecene due to the unique hierarchically porous structure.

Keywords: hierarchically porous; MIL-101(Cr); butyric acid; MO adsorption; catalysis

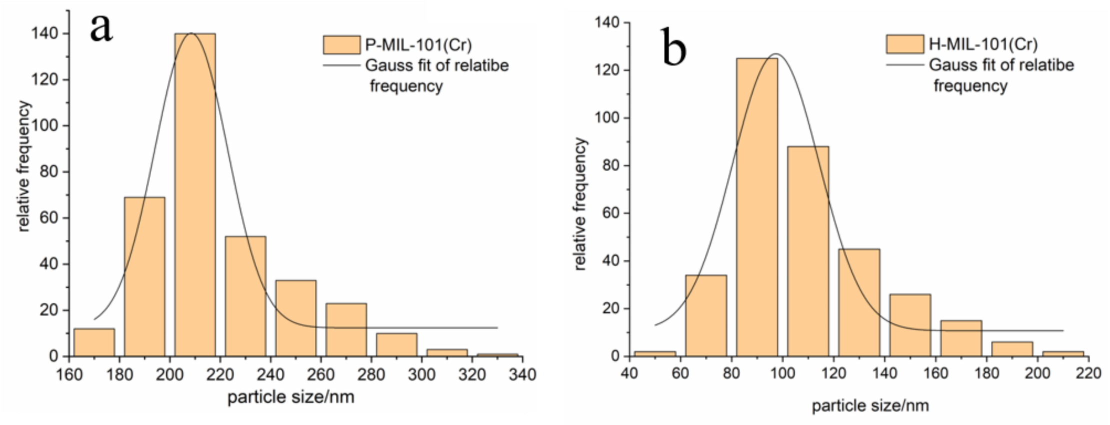

Figure S1. Particle size distribution of P-MIL-101 (Cr) (**a**) and H-MIL-101 (Cr) (**b**).

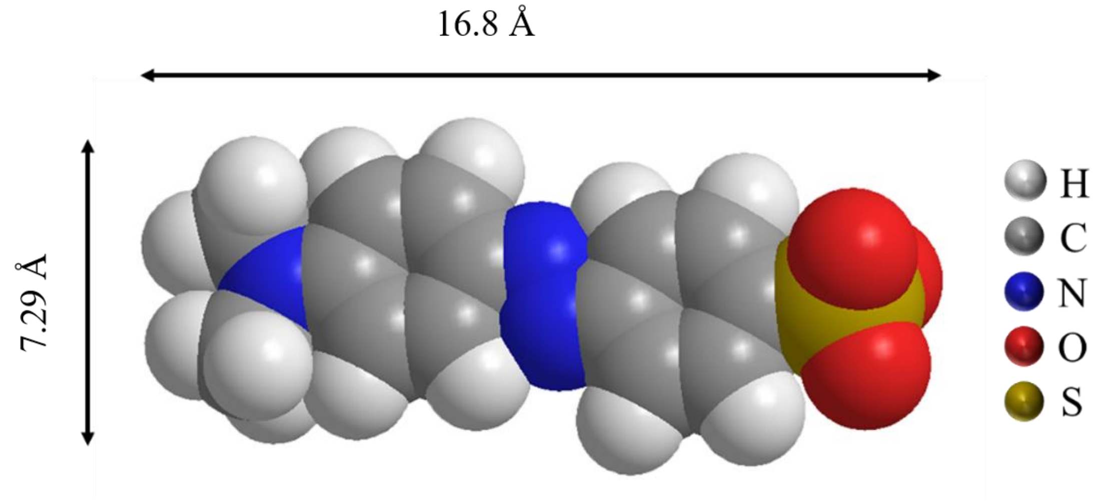

Figure S2. Dye molecule diagram of MO.

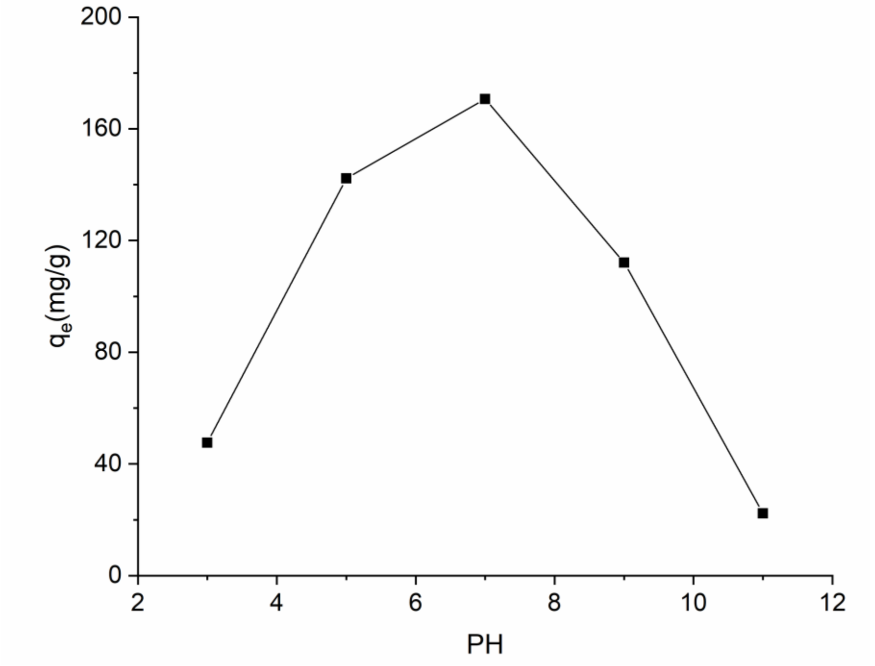

Figure S3. Effect of pH on the adsorption of MO by P-MIL-101

Table S1. Adsorption capacities (q_{max}) of MO on several adsorbents.

Adsorbents	q_{max} (mg g^{-1})	Ref.
Ca/Mg/Al/Fe layered double hydroxides	189.6	[34]
Protonated ED-MIL-101	194	[34]
UiO-66-NH_2	184.4	[34]
ZIF-67 composite	180	[34]
Hyper-cross-linked aromatic triazine porous polymer	249.3	[35]
Hierarchically mesostructured MIL-101	277.8	[35]
Cd-based MOF	167	[35]
GrO@MCr composite	235	[35]
Fe_3O_4@SiO_2@UiO-66	219	[36]
Zwitterionic MOFs@CNF	49.2	[36]
Zn-MOF/chitosan	202	[36]
Nitrogen-doped nanoporous carbon	222.2	[36]
H-MIL-101(Cr)	369.8	This work

Table S2. Characteristic parameters of the adsorption of dyes on the samples.

		Parameter	P-MIL-101(Cr)	H-MIL-101(Cr)
Adsorption kinetics	Pseudo-first-order	$q_{e,Exp}$ (mg g^{-1})	117.4	204.6
		$q_{e,Cal}$ (mg g^{-1})	1.7742	1.4975
		k_1 (min^{-1})	−0.0025	−0.0017
		R^2	0.4493	0.4050
	Pseudo-second-order	$q_{e,Exp}$ (mg g^{-1})	117.4	204.6
		$q_{e,Cal}$ (mg g^{-1})	87.2422	105.9682
		k_2 (g mg^{-1} min^{-1})	0.0082	0.0072
		R^2	0.9999	0.9999
Adsorption isotherm	Tempkin	A (L g^{-1})	0.5002	0.7674
		B	43.7801	53.8791
		R^2	0.9359	0.9675
	Freundlich	$1/n$	0.2553	0.1955
		k_F (mg g^{-1} (L mg^{-1})$^{1/n}$)	51.0604	110.8170
		R^2	0.8856	0.9421
	Langmuir	$q_{m,Exp}$ (mg g^{-1})	229.4	349.8
		$q_{m,Cal}$ (mg g^{-1})	249.1817	369.7633
		k_L (L mg^{-1})	0.0260	0.0385
		R^2	0.9996	0.9999

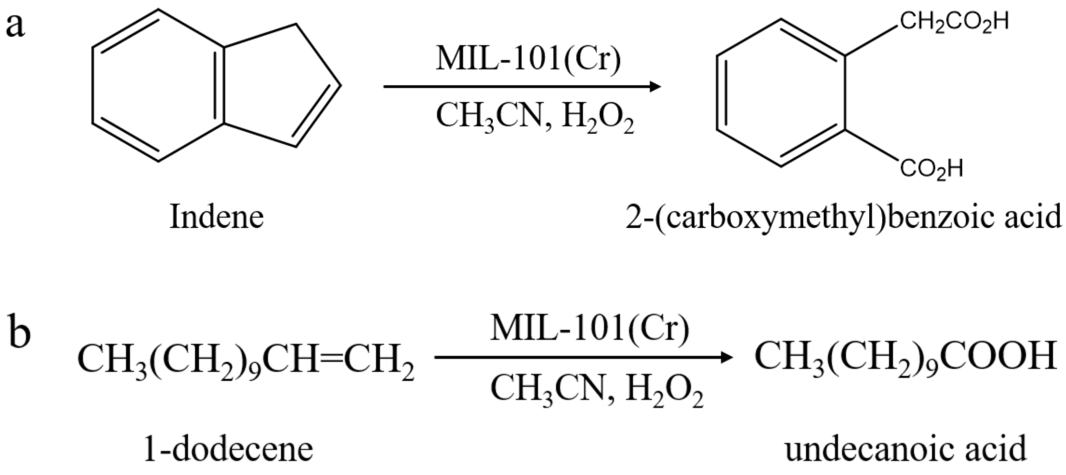

Figure S4. Reaction on scheme for (**a**) indene oxidation reaction, (**b**) 1-dodecene oxidation

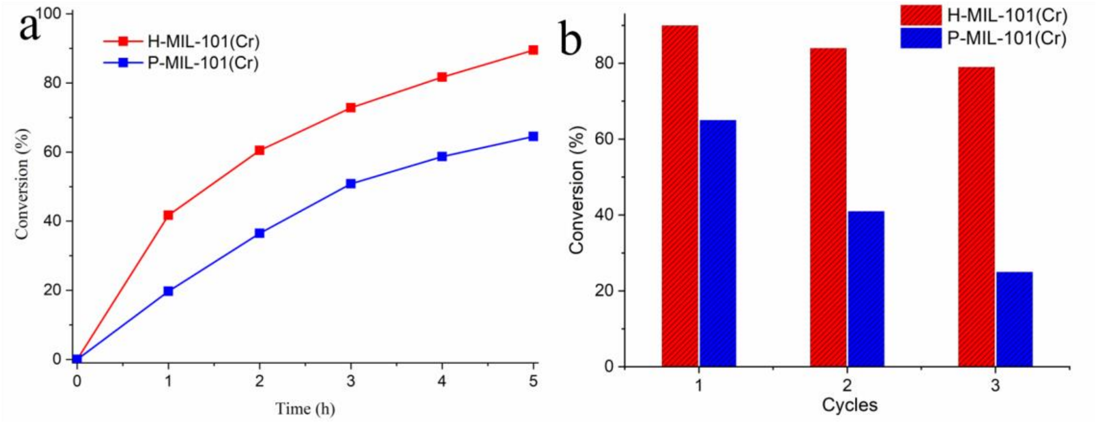

Figure S5. (**a**) Time-dependent conversion of 1-dodecene by P-MIL-101(Cr) and H-MIL-101(Cr). (**b**) Comparison of the conversion for P-MIL-101(Cr) and H-MIL-101(Cr) over three reaction runs, the reaction time is 20 min.

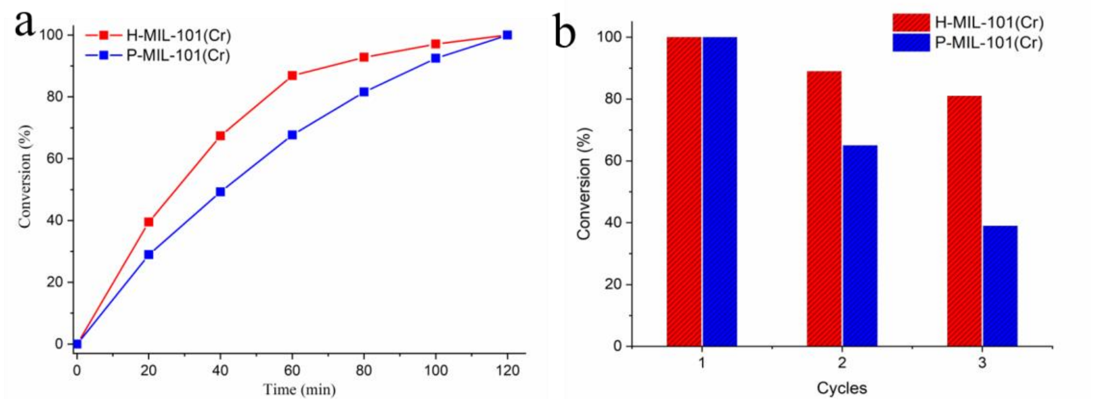

Figure S6. (**a**) Time-dependent conversion of indene by P-MIL-101(Cr) and H-MIL-101(Cr). (**b**) Comparison of the conversion for P-MIL-101(Cr) and H-MIL-101(Cr) over three reaction runs, the reaction time is 20 min.
